# Carbon isotopic evidence for rapid methane clathrate release recorded in coals at the terminus of the Late Palaeozoic Ice Age

**DOI:** 10.1038/s41598-019-52863-6

**Published:** 2019-11-12

**Authors:** Nikola Van de Wetering, Joan S. Esterle, Suzanne D. Golding, Sandra Rodrigues, Annette E. Götz

**Affiliations:** 1 Vale-UQ Coal Geosciences Program, School of Earth and Environmental Sciences, University of Queensland, Brisbane, Queensland 4102 Australia; 2 School of Earth and Environmental Sciences, University of Queensland, Brisbane, Queensland 4102 Australia; 3 Technische Universität Ilmenau, D-98693 Ilmenau, Germany

**Keywords:** Carbon cycle, Carbon cycle, Palaeoclimate, Palaeoclimate

## Abstract

The end of the Late Palaeozoic Ice Age (LPIA) ushered in a period of significant change in Earth’s carbon cycle, demonstrated by the widespread occurrence of coals worldwide. In this study, we present stratigraphically constrained organic stable carbon isotope (δ^13^C_org_) data for Early Permian coals (312 vitrain samples) from the Moatize Basin, Mozambique, which record the transition from global icehouse to greenhouse conditions. These coals exhibit a three-stage evolution in atmospheric δ^13^C from the Artinskian to the Kungurian. Early Kungurian coals effectively record the presence of the short-lived Kungurian Carbon Isotopic Excursion (KCIE), associated with the proposed rapid release of methane clathrates during deglaciation at the terminus of the Late Palaeozoic Ice Age (LPIA), with no observed disruption to peat-forming and terrestrial plant communities. δ^13^C_org_ variations in coals from the Moatize Basin are cyclic in nature on the order of 10^3^–10^5^ years and reflect changes in δ^13^C_org_ of ~±1‰ during periods of stable peat accumulation, supporting observations from Palaeozoic coals elsewhere. These cyclic variations express palaeoenvironmental factors constraining peat growth and deposition, associated with changes in base level. This study also demonstrates the effectiveness of vitrain in coal as a geochemical tool for recording global atmospheric change during the Late Palaeozoic.

## Introduction

The end of the Late Palaeozoic Ice Age (LPIA) represents one of the most extreme climate transformations in geological history, transitioning from icehouse to greenhouse conditions^1–3^. This global event is critical to understanding changes in the carbon cycle associated with the highest rates of global organic carbon burial (up to 6.5 × 10^18^ mol/Myr) in the past half billion years^4^, and coal formation across the terrestrial lithosphere.

The terminal deglaciation of the LPIA was an irregularly distributed, asynchronous event occurring from the Late Palaeozoic, where polar ice melted due to continental scale warming at high-latitudes over Gondwana^1,5,6^. The multiple ice centres of the LPIA across Gondwana are evidenced by widespread glacial deposits in Palaeozoic-aged basins of Australia, Antarctica, South America, Arabia, India and Africa^1,2,7^. Recently, growing evidence suggests that the LPIA may have been triggered, and subsequently terminated, by uplift and erosion of the Hercynian Mountains^8^.

δ^13^C data has been increasingly utilised to understand both palaeoclimatic and palaeoenvironmental changes associated with the LPIA^5,6,9–11^. Recently, a negative isotopic shift in δ^13^C was observed in both inorganic and organic carbon sources, in sediments from high and low latitude Palaeozoic basins^11^. This δ^13^C excursion has been proposed as the Kungurian Carbon Isotopic Excursion (KCIE), and hypothesised to record the release of extremely depleted (ca. −35‰) methane clathrates during glacial melting^11^.

Despite coal occurrence recording the high rates of carbon burial during this period, high-resolution δ^13^C_org_ records of coals deposited during the LPIA have not yet been used to demonstrate changes in the global carbon cycle. The slow accumulation of peat, ~0.9 mm/yr in modern high-latitude settings^12^, allows subsequently formed coals to continuously record palaeoclimate and palaeoenvironmental change at high-temporal resolutions.

This study investigates if the proposed KCIE is evidenced in δ^13^C_org_ of Early Permian coals of the Moatize Basin, Mozambique. These coals were selected for this investigation as they are associated with the final occurrence of widespread glacial deposits, and interbedded with deglacial lacustrine sediments, noted throughout other stratigraphically equivalent basins in Southern Africa^13–16^.

**Figure 1:**
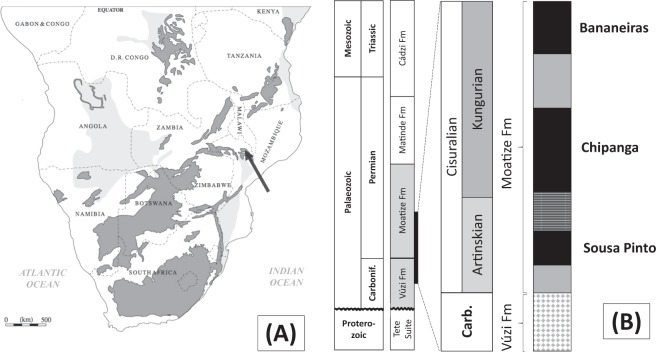
(**A**) Map of Southern Africa^36^, Palaeozoic (Karoo Basin) and stratigraphically equivalent sediments coloured in dark grey, subcrop in light grey, location of Moatize Basin marked by arrow (longitude 16.172772S, latitude 33.806066 E), (**B**) stratigraphy of the Moatize Basin, with inset of simplified stratigraphy of the sampled interval from the Early Permian, Moatize Formation; black indicates coal seams, diamond indicates glacial sediments, grey horizontal pattern indicates lacustrine black shales, grey indicates clastic interburden.

Samples from core recovered by Vale Moçambique were utilised from available core across eight (8) locations in the Moatize Basin, Tete Province, central Mozambique (Fig. 1A). These cores intersect target stratigraphy for the Vale Moçambique mine, that include thick (>10 m) coal accumulations. Coals occur within both the Permian Matinde and Moatize formations (Fig. 1B), although the thickest accumulation of coals is within the lower Moatize Formation (net coal ~52.9 m). Coals were sampled from the lower Moatize Formation that conformably overlies the glacial sediments of the Vúzi Formation marking local glacial to deglacial transition^15^. The glacial sediments and the lacustrine sediments above the first correlatable coal seam, Sousa Pinto seam, to the base of the Chipanga seam, aided in the correlations between drill cores across the basin^15^, in the absence of better chronostratigraphic markers (e.g., volcanic ash).

## Results

**Figure 2:**
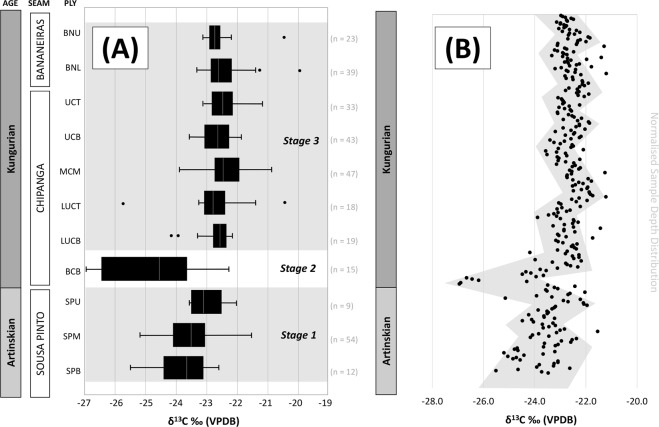
(**A**) Compiled data of Moatize Formation coals by ply domain (SPB - Sousa Pinto base, SPM - Sousa Pinto middle, SPU - Sousa Pinto upper, BCB - basal Chipanga, LUCB - lower Chipanga base, LUCT - lower Chipanga top, MCM - middle Chipanga, UCB - Upper Chipanga base, UCT - Upper Chipanga top, BNL - Bananeiras lower, BNU - Bananeiras upper). (**B**) compiled data of Moatize Formation coals normalised by sample distribution within each ply domain, grey shading highlighting Stage 1/3 δ^13^C cycling and Stage 2 negative δ^13^C excursion.

The range of δ^13^C_org_ for the coals of the Moatize Formation falls within the typical range of C3 plant organic matter^17^, ranging from an absolute maximum of −20.0‰, to an absolute minimum of −26.9‰. The data were collected across all locations domained by each respective ply within the Bananeiras, Chipanga, and Sousa Pinto seams (Fig. 2A). From observing the range of data within these domains, three distinctive ply-domained stages are interpreted.

### Stage 1 – Initiation of peat accumulation

Stage 1 coals, encapsulate the Sousa Pinto seam (plys SPB, SPM, SPU). The average δ^13^C_org_ value for this stage is −23.5‰ (σ = 0.8‰, n = 75), exhibiting a shift to more positive δ^13^C_org_ with time.

Stage 1, Artinskian coals of the Sousa Pinto seam exhibit variable ranges of δ^13^C_org_, suggesting some variation in palaeoenvironmental factors controlling low-magnitude (~±1‰) δ^13^C cycling. A weak (~1.5‰) positive shift in δ^13^C_org_ of Stage 1 coals suggests a more long-lived change in atmospheric CO_2_ concentrations and δ^13^C. These changes are concurrent with the development of widespread peat deposits, resulting in increased rates of carbon burial coincident with the Artinskian^6,8^.

### Stage 2 **–** Terminal deglaciation

**Figure 3:**
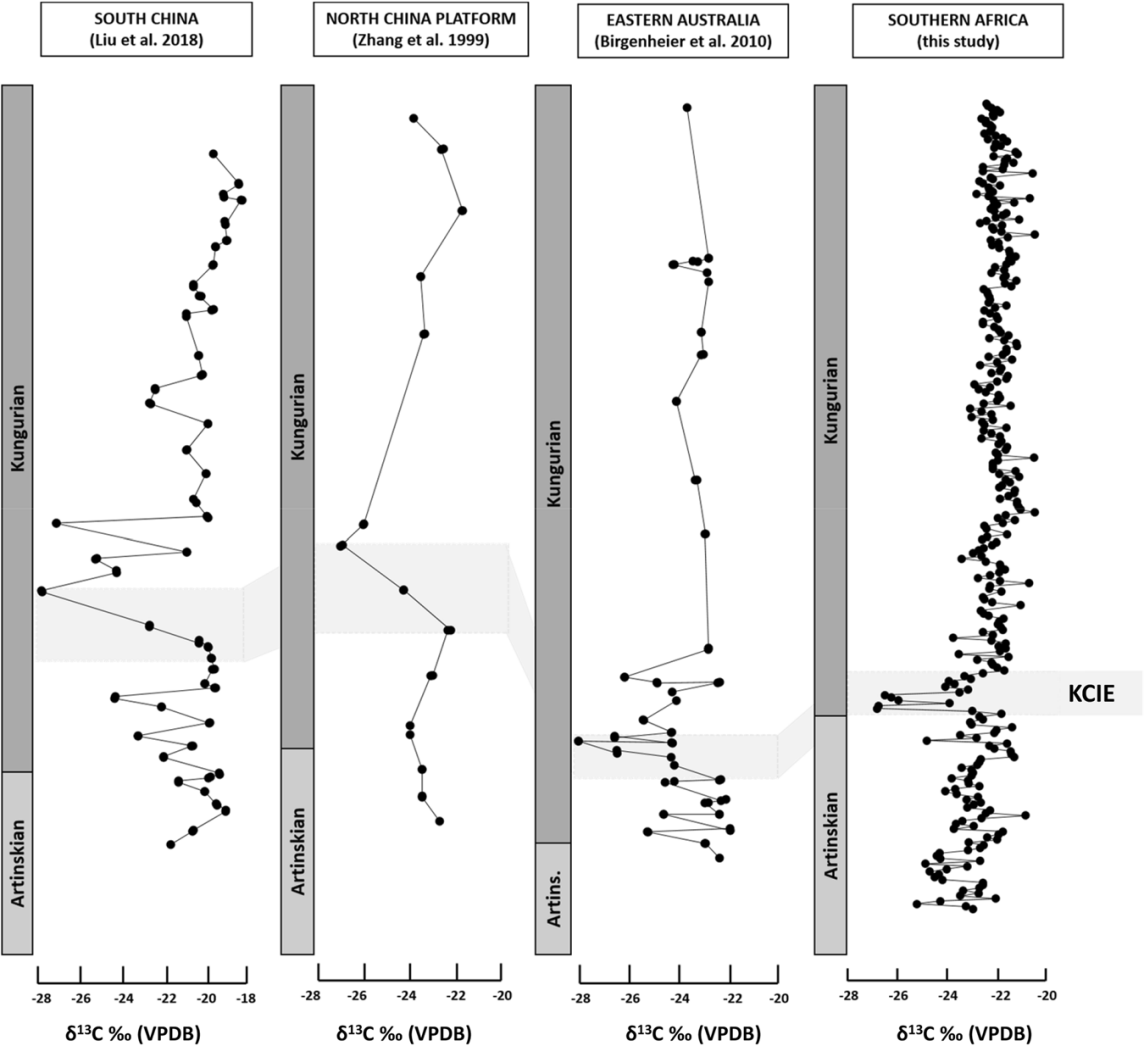
Comparison of geochemical data from other studies^5,11,37^, with this study; grey shading highlighting the proposed Kungurian Carbon Isotopic Excursion (KCIE) interval.

Stage 2 coals, encapsulate the basal Chipanga seam ply only (BCB). The mean δ^13^C_org_ value for this stage is −24.7‰, with a high standard deviation (σ = 1.5‰, n = 15). Stage 2 coals have the most negative δ^13^C_org_ of all the data domains. A striking, high-magnitude (~3.5‰) negative excursion is observed δ^13^C_org_ in Stage 2, coincident with the base of the Chipanga seam in the early Kungurian. This negative excursion is relatively short-lived compared to smaller-scale δ^13^C_org_ cycles (~±1‰) in Stage 1 coals of the Artinskian, and Stage 3 coals of the Kungurian. The compiled δ^13^C_org_ record from the Moatize Formation coals is time equivalent to other, continuous δ^13^C_org_ records from sediments in both low and high-latitude sediments (Fig. 3), suggesting the observed negative carbon shift may be the globally recorded KCIE.

### Stage 3 – Cyclic pluvials

Stage 3 coals, encapsulate the lower Chipanga seam, through to the upper Bananeiras seam (plys LUCB, LUCT, MCM, UCB, UCT, BNL and BNU). The average δ^13^C_org_ value for this stage is −22.6‰ (σ = 0.6‰, n = 222), and remains stable throughout each ply domain, regardless of seam and age distribution. When the total sample set (excluding statistical outliers, n = 305) is normalised for each ply domain, the cyclic variation of δ^13^C_org_ within these coals can be observed (Fig. 2B). Mechanisms for these δ^13^C_org_ cycles are further discussed below.

## Discussion

The Early Permian coals of the Moatize Formation exhibit a three-stage evolution in atmospheric δ^13^C from the Artinskian to the Kungurian. In this study, δ^13^C_org_ cycles (particularly striking in Stage 3, see Fig. 2B), indicate a ~±1‰ shift in δ^13^C_org_, over discrete, regular spacing at normalised depths, from which time intervals may be estimated.

Cyclic variation of δ^13^C_org_ in coal at similar scales has been previously observed in high-resolution isotopic studies from Eastern Australia^18–20^. In these works, the primary control on the distribution of δ^13^C cycles within coal is attributed to palaeoenvironmental factors controlling peat accumulation, including water availability, salinity, pH and atmospheric temperature^21^. However, the timescales over which these cycles occur have not yet been addressed.

The accumulation rates of peat are dependent on both depositional environment, and biological productivity, often genetically linked with peat-forming plant communities^22^. In the Moatize Formation coals of Stage 3, it is demonstrated that both the plant community, and depositional environment controlling peat distribution remained temporally stable to be able to preserve these δ^13^C_org_ cycles. This also implies a state of atmospheric δ^13^C equilibrium, with no significant injections of isotopically heavy or light carbon, nor major changes in CO_2_ concentrations, to disrupt δ^13^C_org_ cycling.

By assuming a relatively constant rate of peat deposition, similar to modern rates of high latitude peat accumulation^12,23^, with peat-to-coal compaction ratios sourced from literature^22,24–26^, a range of potential time scales for each δ^13^C cycle may be calculated (Table 1).

**Table 1:** Approximation of the duration (yrs) of a single δ^13^C cycle observed over ~10 m of coal in Stage 3.

Peat Accumulation Rate (mm/yr)	Coal:Peat Compaction Ratio			
	1:1.2^24^	1:5.7^25^	1:7^26^	1:10^27^
0.9^12,23^	13333	63333	77777	111111
1.3^23^	9231	43846	53846	76923

From these calculations, it is likely that δ^13^C_org_ cycling occurs on a similar scale to short-term (10^3^–10^5^ years) trends inferred from Palaeozoic palaeosol development^27^, and in palaeofloral communities^28^. These short-term changes observed in low-latitude sediments, attributed to pluvials, result in changes in base level. These base-level changes, coincident with δ^13^C_org_ cycling, are also observed in coals from Eastern Australia^18–20^, and the 10^3^–10^5^ year time-frame is coincident with Milankovitch-scale orbital frequencies^28^, also observed in Mesozoic and Cenozoic coals^29,30^.

The observed KCIE is equivalent to the duration of a 10^3^–10^5^ year cycle. The short-lived nature of this isotopic excursion suggests the rapid injection of ^13^C-depleted carbon into the atmosphere, rather than any relatively long-lived changes in CO_2_ concentration. It is possible that this negative carbon isotopic shift is due to the release of methane clathrates (CH_4_) into the atmosphere during terminal deglaciation. Furthermore, the contribution of deep soil organic carbon (SOC) loss and CH_4_ from terrestrial permafrost may also have contributed to widespread δ^13^C perturbation^31^.

The timing of this rapid CH_4_ release is equivalent to the development of euxinic lake deposits across Southern Africa as a result of deglaciation marking the end of the LPIA^13–15^. The stratigraphic equivalent of these euxinic lacustrine deposits is represented by organic rich black shale separating the Sousa Pinto and Chipanga seams, at variable thickness at each sample location (Fig. 1B).

The accumulation of peat, evidenced by the occurrence of the Sousa Pinto seam during Stage 1, implies that more gradual global scale warming and glacial retreat resulting in base-level rise had initiated in the Artinskian, prior to evidence of any catastrophic CH_4_ release. Furthermore, atmospheric CH_4_ injection indicated by the KCIE seems to have little to no observable effect on peat accumulation subsequent to the ultimate terminus of the LPIA, suggesting peat-forming terrestrial ecosystems remained relatively stable during this period.

This estimated time-frame of carbon cycle perturbation during the KCIE is relatively short lived, corresponding to the short residence time of CH_4_ in the atmosphere^32^. This brief time-period of potential methane clathrate release, and subsequently rapid oxidation to CO_2_, is not accompanied by any known mass extinctions, or terrestrial ecosystem catastrophe during the Early Permian^33^.

These observations suggest that whilst CH_4_ release may have contributed to enhanced global warming during the terminus of the Late Palaeozoic Ice Age, the proposed effects of continental weathering and organic carbon burial linked with uplift and subsequent erosion of the Hercynian range demonstrate what maybe a more profound, and long-lived impact on global climate^8^. Additionally, the lack of observable effects on land plant communities despite significant carbon cycle perturbation during the KCIE event further supports the resilience of terrestrial flora to the effects of global scale atmospheric perturbation^34^.

The authors suggest an understanding of the global carbon cycle across geological time may greatly benefit from further research into δ^13^C_org_ from coals.

## Methods

Samples were taken from plys of the Bananeiras (n = 62, average seam thickness = 8.5 m), Chipanga (n = 175, average seam thickness = 31.3 m) and Sousa Pinto (n = 75, average seam thickness = 13.1 m) coal seams (n_total_ = 312). Great care was taken to only sample bright (vitrain) bands from coals, as to minimise δ^13^C_org_ variation with coal lithotype or biochemical composition^18,21,35^. Vitrains were hand-picked at a millimetre scale to avoid any potential carbonate contamination from mineralised cleats. The typical low taphonomic diversity of peat-forming ecosystems^28^, minimises the likelihood of δ^13^C_org_ variation dependent on taxa^9^.

The δ^13^C_org_ values were determined in the Stable Isotope Geochemistry Laboratory (SIGL) at the University of Queensland using a stable isotope ratio mass spectrometer (Isoprime), coupled in continuous flow mode with an elemental analyser (Elementar Cube) (EA-CF-IRMS). Calibration was performed by use of two standards, USGS24 (−16.1‰ δ^13^CPDB) and NAT76H (−29.26‰ δ^13^CPDB), interspersed throughout analytical runs. Each sample was analysed in duplicate, using 50–200 μg of concentrate combusted at 1020 °C in 3.5 mm × 5 mm tin capsules. Any sample with a beam size outside the working range of 1 × 10^−9^ to 9 × 10^−9^ Å, or with a δ^13^C_org_ result variation between duplicates of >0.4‰, was re-analysed, in accordance with laboratory quality control practices. Final data values were normalised and are reported in ‰ VPDB.
